# Association Between Genetic Variant in the Promoter of Pri-miR-34b/c and Risk of Glioma

**DOI:** 10.3389/fonc.2018.00413

**Published:** 2018-09-26

**Authors:** Jinghui Li, Xiaoyu Liu, Yu Qiao, Renli Qi, Shunjin Liu, Jing Guo, Yang Gui, Juanjuan Li, Hualin Yu

**Affiliations:** ^1^Department of Anatomy & Histology and Embryology, Kunming Medical University, Kunming, China; ^2^Second Department of Neurosurgery, First Affiliated Hospital of Kunming Medical University, Kunming, China

**Keywords:** glioma, miR-34b/c, polymorphism, *TP53*, genetic variant

## Abstract

Growing evidence indicates that p53 can regulate the expression of miRNAs, particularly the miR-34 family members, which are described as potential tumor suppressors. Loss of miR-34 suppresses TP53-mediated cell death, whereas over expression of miR-34 induced apoptosis. The study designed to investigate the association between the pir-miR-34b/c rs4938723, TP53 Arg72Pro and the risk of glioma. We genotyped the two polymorphisms in175 glioma patients and 235 healthy controls using polymerase chain reaction-restriction fragment length polymorphism (PCR-RFLP) and DNA sequencing assay. Association analysis showed that the CC genotype of the pir-miR-34b/c rs4938723 was associated with a significantly decreased risk of glioma compared to the TT genotype (CC vs. TT: adjusted OR = 0.43;95% CI, 0.21–0.87,*P* = 0.02). Moreover, a significant association between the patients with glioma and controls was also observed in a recessive model (OR = 0.41; 95% CI, 0.21–0.81, *P* = 0.007). In contrast, the CC genotype of the TP53 Arg72Pro was associated with a significantly increased risk of glioma compared to the GG genotype (CC vs. GG: adjusted OR = 1.73;95% CI, 1.04–2.89,*P* = 0.04), and a significant association between the patients with glioma and controls was also observed in a recessive model (OR = 2.00; 95% CI, 1.26–3.18, *P* = 0.003). These findings suggest that the pri-miR-34b/c rs4938723CC and TP53 Arg72-Pro polymorphisms may be associated with the risk of glioma.

## Introduction

Glioma, a common intracranial malignant tumor in the central nervous system, has a poor prognosis and high mortality (1–4). The incidence of glioma has been sharply increasing worldwide in recent years (5). Surgery remains the main therapeutic method, although other therapies, including targeting therapy, stem-cell rescue and postoperative supplemented, such as radiotherapy and chemotherapy and/or immunotherapy, are also used to maintain the patient's life (5–7). These treatments are not specific for glioma cells, thus they also damage healthy nervous cells leading to a compromise in brain functions, such as cognitive deficits (8). To develop highly efficient therapeutics for glioma, it is critical to identify molecular markers which are closely correlated to the occurrence, progression, and metastasis.

To date, some risk factors have been found to contribute to the pathogenesis of gliomas, such as ionizing radiation, genetic syndromes, dietary and occupational exposures (9, 10). With regard to genetic factors, studies have shown that the absence of gene encoding tumor protein p53 (TP53) is associated with poor prognosis of glioblastoma (11). The TP53, which is situated on chromosome 17p13, has been considered as one of the most common mutations in human cancer (12). The p53 protein plays important roles in basic cell function, such as cell cycle control, apoptosis, senescence, DNA repair, and metabolism (13). p53 regulates the expression of a group of miRNAs, such as miR-34 family members. Dysfunction of miRNAs was commonly found in colorectal, pancreatic, mammary, ovarian, urothelial, and renal cell carcinomas soft tissue sarcomas and glioblastoma (14, 15). In general, the miR-34 family members (miR-34a, miR-34b, and miR-34c) are considered as tumor suppressors. It is reported that miR-34a could not only inhibit glioma stem cell proliferation, self-renewal and migration via targeting sirtuin1 (SIRT1), induce tumor cell apoptosis and differentiation (16), but also inhibit glioma cells progression and chemo resistance via targeting programmed death ligand 1 (PD-L1) (17). Moreover, miR-34c was proved to be effective in mitigating the cell proliferation, cell cycle changes, apoptosis and cell invasion of glioma cells (5).

Single nucleotide polymorphisms (SNPs) refer to DNA sequence polymorphisms at the genomic level, which occurs in both coding and non-coding regions of genes. SNPs in the promoter regions and coding regions are associated with susceptibility of malignancy. The rs4938723C/T polymorphism in the promoter region of pri-miR-34b/c was predicted to influence the binding of transcription factor GATA-X to its target genes, and thus affect the expression levels of the target genes related to tumor differentiation and tumorigenesis (18–20). Deficiency of miR-34 results in impairment of cell death mediated by TP53, nevertheless, excessive levels of miR-34 induces cell apoptosis (21, 22). Among the genetic variants of the TP53, the most widely studied polymorphism is named Arg72Pro, which is located in the proline-rich domain of the p53 protein (23). Above all, members of the miR-34 family is considered to be potential inhibition of tumor may also be a cause of cancer genes. In this study, we carried out a case–control study to evaluate whether pri-miR-34b/c rs4938723 and TP53 Arg72Pro are associated with the risk of glioma.

## Subjects and methods

### Study populations

The study was ratified by the ethics committee of Kunming Medical University, and written informed consent was obtained from all subjects participating in this study. The case-control study population consisted of 410 unrelated Chinese Han individuals including 175 patients (101 males and 74 females, age: 41.2 ± 16.3 years) newly diagnosed with glioma and 235 controls (156 males and 79 females, mean age: 49.9 ± 11.3 years) living in Yunnan province in China. Patients who diagnosed as glioma were recruited from the First Affiliated Hospital of Kunming Medical University during a period from January 2012 to September 2015.The control group consisted of 235 healthy controls in the same hospital during the same period. Subjects with any disease in nervous system were excluded from this study.

### Genotyping

Approximately 2 ml of venous blood sample was collected from 175 glioma patients and 235 controls. Genomic DNA was extracted using a DNA extraction kit (Bioteke, Beijing, China) on the basis of instructions provided by the manufacturer. The pri-miR-34b/c rs4938723 and TP53 Arg72Pro were genotyped by polymerase chain reaction-restriction fragment length polymorphism (PCR-RFLP) assay. The primer sequences for rs4938723 were as follows: 5′-CCTCTGGGAACCTTCTTTGACCAAT-3′ (sense) and 5′-TGAGATCAAGGCCATACCATTCAAGA-3′ (antisense). The primers for TP53 Arg72Pro were 5′-CCCCCTTGCCGTCCCAAGCAATGG-3′ (sense) and 5′-CTGCTGGTGCAGGGGCCGCG-3′ (antisense). For quality control, all gel pictures were read by two researchers double-blindly, and discrepant results were analyzed repeatedly. Moreover, all the results of PCR-RFLP which confirmed by direct sequencing were coincident.

## Statistical analysis

Hardy–Weigliomaerg equilibrium (HWE) was analyzed by χ^2^ test for each polymorphism among control subjects. The genotype frequencies of pri-miR-34b/c rs4938723 and TP53 Arg72Pro in patients with glioma and controls were compared using χ^2^ test (for categorical variables). A 5% level of significance was used in the analysis, and all statistical tests were two sided. Logistic regression analysis was performed to assess the association of pri-miR-34b/c rs4938723 and TP53 Arg72Pro with the risk of glioma by computing odds ratio (OR) and 95% confidence interval (CI). Statistical analysis of data was performed by SPSS 19.0 statistical software (SPSS Inc., Chicago, IL, USA).

## Results

A total of 175 glioma patients and 235 incognito controls were registered in our study. Table 1 summarizes demographic information and other parameters for all topics. No gender differences were detected between glioma patients and controls. The prevalence of the genotype distributions of pri-miR-34b/c rs4938723 and *TP53* Arg72Pro in patients with glioma and controls was determined to estimate the relationship to the risk of glioma. The genotype and allele frequencies distribution of both polymorphisms in the controls met the requirements of the HWE (*p* > 0.05). Tables 2–4 summarize the genotype and allele frequencies and the combined genotypes frequencies of the pri-miR-34b/c rs4938723 and *TP53* Arg72Pro. Compared to the pri-miR-34b/c rs4938723TT genotype, the CC genotype was found to decrease the risk of glioma (CC vs. TT: OR = 0.43; 95% CI, 0.21–0.87, *P* = 0.02). Moreover, a significant association was found in a recessive model as well (OR = 0.41; 95% CI, 0.21–0.81, *P* = 0.007). In contrast, for *TP53* Arg72-Pro, the CC genotype was associated with a significantly increased risk of glioma compared to the GG genotype (CC vs. GG: OR = 1.73; 95% CI, 1.04–2.89, *P* = 0.04). In addition, a significant association between glioma group and control group was detected in a recessive model (OR = 2.00; 95% CI, 1.26–3.18, *P* = 0.003) (Table 2). However, no significant association was found between pri-miR-34b/c rs4938723 and *TP53* Arg72Pro and the risk of glioma for allelic association analysis (Table 3). Combined analysis of the pri-miR-34b/c rs4938723 and *TP53* Arg72-Pro polymorphisms showed no significant effect on glioma risk (Table 4).

**Table 1:** Characteristics of the study population.

**Variables**	**Patients with glioma (*n* = 175)**	**Controls (*n* = 235)**
**GENDER (%)**		
Male	101 (57.7%)	156 (66.4%)
Female	74 (42.3%)	79 (33.6%)
Age (years, mean ±SD)	41.2 ± 16.3	49.9 ± 11.3
**TUMOR SIZE (%)**		
≤5 cm	115 (65.7%)	
>5 cm	60 (34.3%)	
**CLINICAL STAGES (%)**		
I–II	86 (49.1%)	
III–IV	89 (50.9%)	

*SD, standard deviation*.

**Table 2:** The genotype frequencies of miR-34b/c rs4938723 and TP-53 Arg72Proin glioma patients and controls.

**Polymorphisms**	**Controls (*n* = 235, %)**	**Patients with glioma (*n* = 175, %)**	**OR (95%CI)**	***P*-value**
**rs4938723**				
TT	102 (43.4)	80 (45.7)	1.0 (ref)	
CT	97 (41.3)	83 (47.4)	1.09 (0.72–1.65)	0.68
CC	36 (15.3)	12 (6.9)	0.43 (0.21–0.87)	0.02
Dominant	133 (56.6)	95 (54.3)	0.91 (0.61–1.35)	0.64
Recessive	199 (84.7)	163 (93.1)	0.41 (0.21–0.81)	0.007
***TP*****-53 Arg72Pro**				
GG	92 (39.1)	67 (38.3)	1.0 (ref)	
CG	101 (43.0)	55 (31.4)	0.75 (0.47–1.18)	0.21
CC	42 (17.9)	53 (30.3)	1.73 (1.04–2.89)	0.04
Dominant	143 (60.9)	108 (61.7)	1.04 (0.69–1.55)	0.86
Recessive	193 (82.1)	122 (69.7)	2.00 (1.26–3.18)	0.003

*OR, Odds ratio; CI, confidence interval*.

**Table 3:** The allele frequencies of miR-34b/c rs4938723 and TP-53 Arg72Proin glioma patients and controls.

**Polymorphisms**	**Controls (2*n* = 470, %)**	**Patients with glioma (2*n* = 350, %)**	**OR (95%CI)**	***P*-value**
**rs4938723**				
T	301 (64.0)	243 (69.4)	1.0 (ref)	
C	169 (36.0)	107 (30.6)	0.78 (0.58–1.05)	0.11
***TP*****-53 Arg72Pro**				
G	285 (60.6)	189 (54.0)	1.0 (ref)	
C	185 (39.4)	161 (46.0)	1.31 (0.99–1.74)	0.06

*OR, Odds ratio; CI, confidence interval*.

**Table 4:** Combined effect of miR-34b/c rs4938723 and TP-53 Arg72Proon glioma risk.

**Polymorphisms**	**Controls (*n* = 235, %)**	**Patients with glioma (*n* = 175, %)**	**OR (95% CI)**	***P*-value**
rs4938723TT+*TP53*GG	50 (21.3)	33 (18.9)	1.0 (ref)	
rs4938723CT/CC+*TP53*CG/CC	91 (38.7)	61 (34.9)	1.02 (0.59–1.75)	0.96
rs4938723TT+*TP53*CG/CC	52 (22.1)	47 (26.9)	1.37 (0.76–2.47)	0.30
rs4938723CT/CC+*TP53*GG	42 (17.9)	34 (19.4)	1.23 (0.65–2.30)	0.53

*OR, Odds ratio; CI, confidence interval*.

## Discussion

We conducted a case-control study to evaluate whether the pri-miR-34b/c rs4938723 and *TP53* Arg72Pro polymorphisms influence the susceptibility to glioma in the Han Chinese population and we found that individuals with the CC genotype of pri-miR-34b/c rs4938723 had a lower risk of glioma than the TT genotype. In contrast, individuals with the CC genotype of *TP53* Arg72-Pror had a higher risk of glioma than the GG genotype. These results suggest that the pri-miR-34b/c rs4938723 and *TP53* Arg72Pro may exert different actions in the oncogenesis of glioma.

It is well known that *TP53* and pri-miR-34b/c may singly and/or jointly contribute to the tumorigenesis (24). It was reported that miR-34 suppresses carcinogenesis by repressing murine double minute 4 (HDM4), which is a high-handed negative moderator of p53 (25). Thus, miR-34 could regulate p53 through a positive feedback loop (25). Down-regulation of miR-34a/b/c suppressed tumor formation in colorectal cancer (26), while over-expression of miR-34a in aggressive prostate cancers (PCAs) cells reduced proliferation and colony formation by CtBP1\miR-34a\STMN1\GDF15 pathway (27). Moreover, the polymorphisms of pri-miR-34 b/c rs4938723 have different associations in different types of cancer. Pri-miR-34b/c rs4938723 polymorphism is reported to be related to a decreased risk of childhood acute lymphoblastic leukemia (ALL) (28), gastric Cancer (29) and esophageal squamous cell carcinoma (30). In contrast, Chun-Jia Liu et al. reported that the CC genotype of pri-miR-34b/c rs4938723 had a higher risk of developing hepatocellular carcinoma (31), and it is reported that pri-miR-34b/c rs4938723 polymorphism also increased the risk of prostate cancer (32) and cervical cancer (33). Additionally, it was reported that miR-34 loci methylation was related to the increased risk of non-small-cell lung cancer (34), possibly by altering methylation status of miR-34b/c to affect p53 expression, similar to the mechanism by which miR-34b/c rs4938723 polymorphism affect p53 expression and carcinogenesis of glioma. MiR-34 is reported to inhibit glioma cell proliferation, invasion, self-renewal, migration, and induce tumor cell apoptosis and differentiation (5, 16, 17). The results of our study also showed that pri-miR-34b/c rs4938723 reduced the risk of glioma, indicating that pri-miR-34b/c rs4938723 may exert a protective effect on the incidence of glioma.

p53 inhibits the occurrence and development of cancer effectively (35). So far there have been a lot of experiments to study the relationship between *TP53* Arg72Pro polymorphism and the risk of different kinds of cancer. A meta-analysis conducted by De-Ke Jiang et al. concluded that *TP53* Arg72 carriers can significantly reduce the risk of esophageal cancer (36). In contrast, Dimas-Gonzalez et al. reported that p53 protein was up-regulated in breast cancer (37). The *TP53* Arg72Pro CC genotype may contribute to an increased risk of CRC, especially for rectal cancer and among Asians (38). In this study, we also found that carriers with the *TP53* Arg72-Pro CC genotype will significantly increase the risk of glioma. However, no significance was detected after combining with miR-34b/c rs4938723, implying that pri-miR-34b/c rs4938723 and *TP53* Arg72Pro may be offset each other on the development of glioma.

There are some limitations that should be addressed in this study. First, the small sample size in this study may have an impact on statistical power. Second, follow-up data was not acquired which prevented our further survival analysis. Finally, the study subjects were all ethnic Han Chinese, which raised possibility that our results cannot be used directly to other populations. To address the concern, further large-scale studies among different populations are needed.

In conclusion, our study provides evidence that the pri-miR-34b/c rs4938723 might be a protective factor via decreasing the risk of glioma. On the contrary, *TP53* Arg72-Pro may be a risk factor for glioma. Due to several limitations as discussed above, multi-racial population studies and functional analysis of rs4938723 polymorphism and *TP53* Arg72-Proin gliomas are warranted. Identification of glioma susceptibility genes is a critical step toward a better understanding the underlying mechanisms of glioma carcinogenesis, potentially leading to development of novel, genetic markers and surveillance programs.

## Availability of data and materials

The datasets during and/or analyzed during the current study are available from the corresponding author on reasonable request.

## Author contributions

JuL and HY designed the study. JiL and XL was a major contribute or in writing the manuscript. JiL and XL carried out the molecular genetic studies, participated in the sequence alignment. YQ, RQ, SL, YG, and JG participated in the sequence alignment and performed the statistical analysis. All authors read and approved the final manuscript.

### Conflict of interest statement

The authors declare that the research was conducted in the absence of any commercial or financial relationships that could be construed as a potential conflict of interest.
